# A case report of X-linked centronuclear myopathy in a neonate: clinical presentation, therapeutic process, and genetic insights

**DOI:** 10.3389/fped.2026.1889549

**Published:** 2026-07-14

**Authors:** Huan Liao, Mengyuan Jiang, Xinyan Zhong, Xiaohua Luo, Hongsheng Qiu, Hua Pan, Jinghui Gan, Shibing Zhong

**Affiliations:** 1 Neonatal Department, Ganzhou Women and Children's Health Care Hospital, Zhanggong District, Ganzhou, Jiangxi, China; 2 Medical Genetics Department, Ganzhou Women and Children's Health Care Hospital, Zhanggong District, Ganzhou, Jiangxi, China; 3 Army Medical University, Chongqi, China

**Keywords:** asphyxia, genetic testing, MTM1 gene, neonate, x-linked centronuclear myopathy (XLCNM)

## Abstract

X-linked centronuclear myopathy (XLCNM) is a rare neuromuscular disorder that poses significant diagnostic and treatment challenges, particularly in neonates. We present a case of a male infant born at 36 weeks of gestation, weighing 2.1 kg, who was admitted 51 min after birth due to severe asphyxia and low birth weight. Following an emergency cesarean section, the infant required resuscitation, including tracheal intubation and medication administration. Initial evaluations indicated severe respiratory distress and low muscle tone, along with various clinical abnormalities. Laboratory tests suggested potential infection and metabolic distress. Despite comprehensive intensive care, including mechanical ventilation, anti-infection regimens, and nutritional support, the infant continued to experience respiratory difficulties without improvement. Genetic testing via whole exome sequencing identified a rare variant in the *MTM1* gene, confirming the diagnosis of XLCNM with an X-linked recessive inheritance pattern. Unfortunately, after extensive treatment and family discussions, the infant's condition failed to stabilize, leading to the difficult decision to withdraw therapy, resulting in the patient's passing. This case underscores the urgent need for early recognition of XLCNM and the critical role of genetic counseling for affected families.

## Introduction

Congenital myopathies (CM) represent a group of rare inherited muscle disorders characterized by a considerable range of clinical and genetic heterogeneity. Among these disorders, one noteworthy subtype is central nuclear myopathy (CNM), which has garnered attention due to its significant clinical implications and complex genetic foundations (1). Centronuclear myopathy, commonly referred to as myotubular myopathy, can be categorized into three distinct types based on the underlying pathogenic genes, modes of inheritance, and age at onset: X-linked centronuclear myopathy (XLCNM), centronuclear myopathy type 1, and centronuclear myopathy type 2 (2). Despite the advancements in research, these conditions remain difficult to diagnose and manage, and effective therapeutic options are still limited (3).

The rarity and genetic variability of congenital myopathies, including CNM, result in a diverse array of clinical presentations, histopathological features, and genetic profiles (4). Specifically, CNM is a hereditary muscle disorder with an estimated incidence of about 1 to 2 cases per 100,000 individuals (5). Of particular importance is X-linked centronuclear myopathy, which stems from mutations in the MTM1 gene and affects approximately 1 in 50,000 males (6). The MTM1 gene encodes myotubularin, an endosomal phosphatase that plays a crucial role in dephosphorylating key second messengers, such as phosphatidylinositol 3-phosphate (PI3P) and inositol 1,3,5-trisphosphate (InsP3).

Clinically, XLCNM is characterized by profound muscle weakness, leading to significant disabilities, including dependence on ventilatory support and wheelchair use. Unfortunately, this condition is often associated with early mortality among affected individuals (7). Given the intricate nature of XLCNM, early identification and diagnosis are paramount. Therefore, clinicians encountering neonates with unexplained low muscle tone, excessive secretions, and respiratory dependency must consider genetic testing as an essential diagnostic tool.

In view of the complexities inherent to XLCNM, this report details the case of a neonate diagnosed with severe asphyxia, who was later found to have X-linked centronuclear myopathy through genetic testing during hospitalization. We will discuss the clinical presentation, management strategies, and the significance of genetic insights in enhancing diagnostic accuracy and informing treatment decisions in similar cases.

## Case presentation

### Clinical basic information

Patient, male, gestational age 36 weeks and 3 days, birth weight 2.1KG. The chief complaint is due to premature birth, low birth weight, and asphyxia during delivery. The patient was admitted to the hospital 51 min after birth. The mother reported that she had regular prenatal check-ups during the early and mid-pregnancy with no abnormalities, and she had gestational diabetes in the late pregnancy, which was controlled through diet. At 36 weeks of gestation, an ultrasound scan showed an amniotic fluid index of 447. Therefore, she was admitted to the obstetrics and gynecology department for dexamethasone treatment to promote fetal lung maturity. Half an hour before delivery, vaginal discharge occurred, approximately 2,000 ml in volume, accompanied by irregular lower abdominal distension pain. The fetal heart rate monitoring indicated frequent decelerations, suggesting fetal distress, possible placental abruption, and high-risk factors for polyhydramnios. An emergency cesarean section was performed under tracheal intubation and general anesthesia in the lower segment of the uterus to deliver the fetus.At birth, the amniotic fluid was clear. Immediate physical examination revealed poor neonatal responsiveness, generalized cyanosis, absence of spontaneous crying, no muscle tone in limbs, a heart rate of approximately 40 beats per minute on auscultation, and no effective spontaneous breathing—consistent with severe neonatal asphyxia. Neonatal resuscitation was immediately initiated according to standard protocols, with specific time points and interventions as follows:Immediately after delivery: Vital signs were assessed—heart rate 40 bpm, no spontaneous breathing, cyanotic skin color, no response, and absent muscle tone. Airway was cleared promptly, followed by successful endotracheal intubation. Positive pressure ventilation via endotracheal tube was started immediately, along with synchronized chest compressions. Umbilical venous access was established, and advanced resuscitative measures were initiated.

At 30 s after birth, the heart rate was 50 bpm; 0.6 ml of 1:10,000 epinephrine hydrochloride was administered intravenously via umbilical vein. At 60 s post-birth, the heart rate remained at 54 bpm, with persistent cyanosis and poor responsiveness; an additional 0.3 ml of 1:10,000 epinephrine hydrochloride was given via umbilical vein. Immediately afterward, 20 ml of normal saline was slowly infused (over 10 min) through the umbilical vein for volume expansion.Apgar score at 1 min: 2 points (heart rate 1 point, response to stimuli 1 point; respiratory effort, skin color, and muscle tone 0 points).At 1 min and 30 s after birth, the heart rate increased to 110 bpm, indicating recovery above 100 bpm. Chest compressions were stopped immediately, and positive pressure ventilation via endotracheal tube was continued to support breathing.At 2 min after birth, the heart rate was 114 bpm, but the infant showed poor responsiveness, weak spontaneous breathing, improved skin color, and low limb tone. Mechanical ventilation via endotracheal tube was continued.At 5 min after birth, the infant's condition was reassessed under continuous ventilator support. The 5 min Apgar score was 6 points (respiratory effort 1 point, heart rate 2 points, skin color 2 points, response to stimuli 1 point, muscle tone 0 points).This infant was a high-risk cesarean delivery newborn with multiple antenatal risk factors and severe perinatal asphyxia. Despite improvement in heart rate and skin color following aggressive resuscitation, the infant exhibited weak spontaneous breathing and poor responsiveness, indicating critical illness and ongoing risks of unstable cardiorespiratory function. The infant was maintained on endotracheal intubation with continuous positive pressure ventilation, closely monitored by obstetric and neonatal care teams, and safely transferred to the neonatal intensive care unit for further management.

Upon admission, vital signs revealed a temperature of 36.5°C, pulse of 146 bpm, respiration of 45 breaths/min, oxygen saturation of 85%, and weight of 2,100 g. The preterm infant demonstrated poor responsiveness, rapid breathing, and cyanosis, with key neurological and physical assessments indicating low muscle tone and weak reflexes. The father is a healthy 36-year-old with blood type B, while the mother, also 36, has gestational diabetes and blood type B, with a stable glucose control history. The maternal obstetric history indicates G2P2 status, and the patient has a 7-year-old healthy sister.

### Supplementary examinations

**Figure 1:**
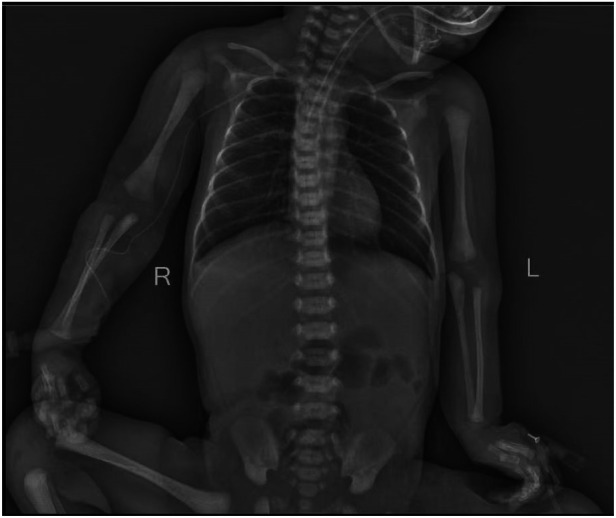
The x-ray chest examination results of the proband.

**Figure 2:**
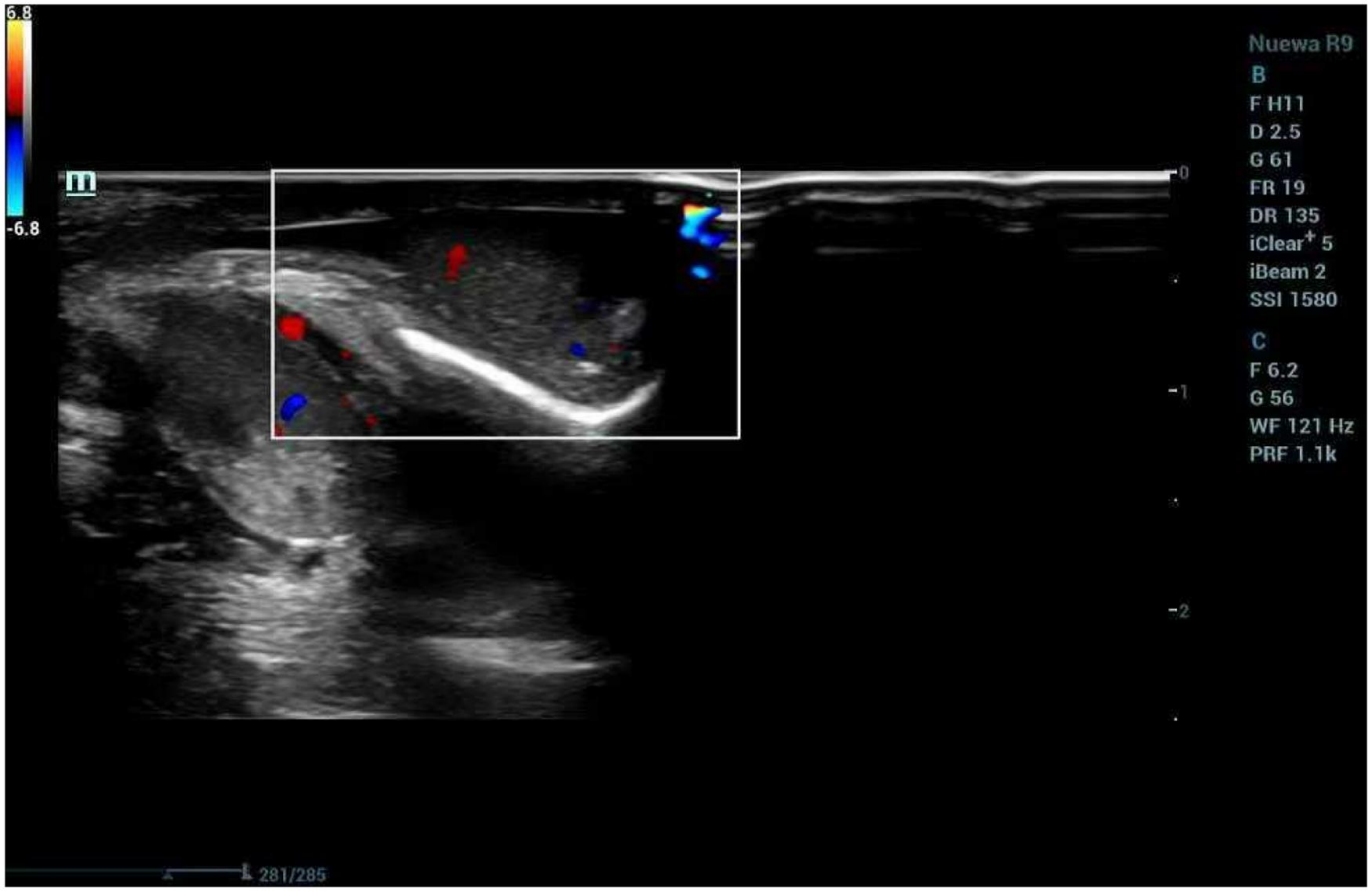
The results of testicular ultrasound in the proband.

**Figure 3:**
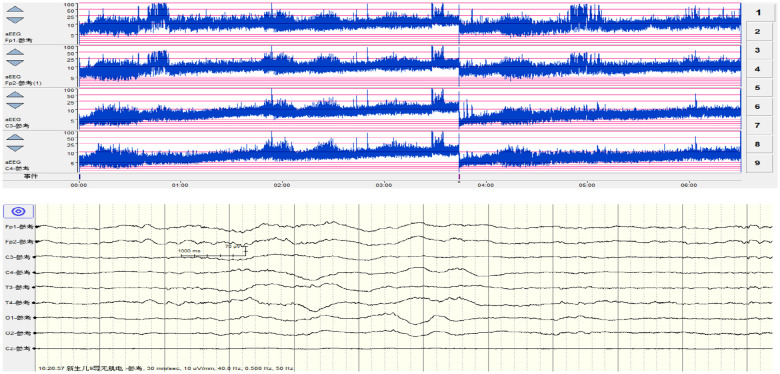
The results of amplitude-integrated electroencephalogram in the proband.

Post-admission laboratory tests revealed a white blood cell count of 14.19 × 10^9^/L, with a neutrophil percentage of 56.4%. Hemoglobin was measured at 173 g/L, and hematocrit at 52.7%. C-reactive protein was low (0.01 mg/L). Blood gas analysis indicated a pH of 7.074, partial pressures of oxygen and carbon dioxide of 95.3 mmHg and 96.2 mmHg, respectively, with oxygen saturation at 92.6% and lactate at 1.22 mmol/L. Liver function tests showed total bilirubin at 48.0 µmol/L (direct 20.1 µmol/L, indirect 27.9 µmol/L), total protein at 45.20 g/L, and albumin at 32.60 g/L. Myocardial enzymes displayed creatine kinase at 112 U/L and CKMB at 9.60 ng/ml. Renal function and electrolytes were normal. Coagulation tests indicated activated partial thromboplastin time at 45.08 s and fibrinogen at 1.82 g/L. Thyroid function tests showed thyroid-stimulating hormone at 5.380 µIU/mL, free triiodothyronine at 4.770 pmol/L, and free thyroxine at 22.800 pmol/L. Blood cultures were negative for bacteria and fungi, and sputum culture was normal. Chromosome analysis revealed a normal karyotype (46,XY). Blood type was B, RH(D) positive, with no irregular antibodies. Chest x-ray shows no obvious abnormalities in the heart, lungs, or diaphragm, and no signs of elevated diaphragm are present (Figure 1). Color Doppler ultrasound of the head showed no significant abnormalities, while lung ultrasound revealed partial consolidation of the right upper lobe. Echocardiography indicated an enlarged right heart with left-to-right shunt (5.1 mm), a patent ductus arteriosus (2.5 mm), mild tricuspid regurgitation, and intact left heart function. Abdominal ultrasound showed no significant lesions in the liver, gallbladder, spleen, pancreas, or kidneys. Testicular ultrasound indicated low echogenicity in the left groin, suggestive of cryptorchidism, and hydrocele in the right testicular sheath (Figure 2). Amplitude-integrated electroencephalogram revealed no significant abnormalities (Figure 3).

### Treatment and outcome

Upon admission, the infant received invasive ventilator-assisted breathing, anti-infection treatment, caffeine citrate for respiratory stimulation, and mild hypothermia therapy. From August 4th to 6th, the child displayed active spontaneous breathing and was transitioned to non-invasive ventilation. However, on the night of August 6th, weak spontaneous breathing and low blood oxygen saturation necessitated a return to invasive ventilation. The anti-infection regimen was adjusted based on pulmonary infection and inflammatory markers, administering cefoperazone-sulbactam, amoxicillin-clavulanate, and ceftazidime along with ampicillin for infection control and fluconazole for fungal prevention.Due to weak sucking ability, nasogastric feeding of formula/breast milk was initiated, supplemented with intravenous nutrition. Continuous aspiration was performed to manage excessive secretions. Albumin was infused for hypoproteinemia, red blood cell transfusions were given to address anemia, gangliosides were administered for brain nourishment, and phototherapy was utilized for jaundice. Despite these interventions, the child showed no significant improvement in muscle tone or secretions and remained dependent on invasive ventilation.

After admission and active treatment, extubation failed, with low muscle tone and excessive secretions, and the condition showed no significant improvement. Could there be another underlying disease? Differential diagnosis 1: Congenital muscular dystrophy. Clinical features include a hereditary muscular disorder characterized by very early-onset muscle weakness, presenting as progressive symmetric muscle weakness and atrophy, ultimately leading to complete loss of motor function, typically manifesting in early life, infancy, or shortly after birth. Facial muscles, respiratory muscles, and swallowing function are often affected. Inheritance pattern: primarily autosomal recessive. Diagnosis: In newborns, plasma CK levels are usually below 200 IU/L; significantly elevated levels are an important diagnostic criterion for congenital muscular dystrophy. Genetic testing and, when necessary, muscle biopsy can confirm the diagnosis.Differential diagnosis 2: Congenital myasthenic syndrome. This is a group of inherited neuromuscular junction disorders causing myopathy. In newborns, it may present with muscle weakness, poor crying, ocular fatigue after activity, and respiratory insufficiency. It responds to anticholinesterase drugs, shows positive Tensilon test results, exhibits decremental response on low-frequency repetitive nerve stimulation on electromyography, and displays repetitive mixed motor unit action potentials on single stimulation. Diagnosis is confirmed by genetic testing.Differential diagnosis 3: Congenital myopathy. This is a group of rare inherited myopathies. Typical early clinical manifestations include hypotonia (“limp infant syndrome”), muscle weakness, malnutrition, and/or delayed motor milestones, often accompanied by respiratory and/or bulbar involvement. Diagnosis is typically established through genetic testing.

To further clarify the diagnosis, on the day of admission, we had a conversation with the parents of the child and suggested that they complete the genetic test. However, the parents did not agree. After repeated communication, the parents finally agreed on the 10th day of the child's hospitalization and completed the whole exome sequencing analysis for the child. After the results of the whole exome gene sequencing of the child were returned on the 40th day of hospitalization, Telemedicine consultation was arranged with the Department of Neurology, Peking University First Hospital to facilitate comprehensive clinical assessment of the neonate. They then gave the child oral administration of salbutamol sulfate tablets (1/8 tablet, twice a day), pyridostigmine bromide (1/8 tablet, once every 8 h), and tamoxifen tablets (1/8 tablet, once a day). After 2 weeks of oral medication treatment, the child still could not be weaned off the invasive ventilator, the secretions did not decrease, and the effect was poor. Under the active treatment of breathing, nutrition, and maintaining the stability of the internal environment, the weaning from the ventilator failed. The muscle tone remained low, and the secretions were abundant. The condition did not show significant improvement. The treatment continued until September 23rd when the family gave up the treatment and the child died.

**Figure 4:**
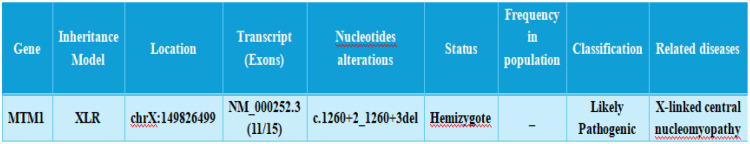
The results of the whole exome sequencing of the proband.

**Figure 5:**
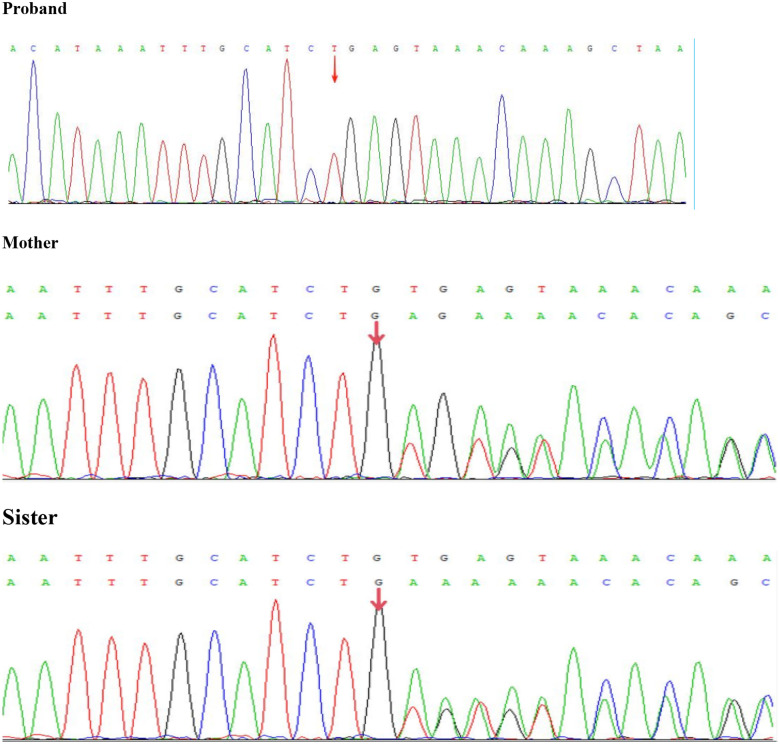
The direct sequencing results of the proband, and her mother and sister.

The subsequent findings indicated an X-linked recessive inheritance pattern with mutations in the *MTM1* gene, which may lead to X-linked central nucleomyopathy. A rare frameshift mutation was identified at the canonical splicing site within intron 11 of the *MTM1* gene, categorized as possibly pathogenic according to ACMG guidelines (Figure 4). Carrier status verification through Sanger sequencing for the mother and sister confirmed both as heterozygous carriers of the same mutation, consistent with the X-linked recessive inheritance pattern (Figure 5).

## Discussion

We present a case of a male late preterm infant diagnosed with X-linked centronuclear myopathy (XLMTM), characterized by low muscle tone, excessive secretions, and inability to wean from invasive ventilation. XLMTM exhibits significant phenotypic variability; in its most severe forms, affected males experience profound muscle weakness, hypotonia, extraocular muscle paralysis, and respiratory failure during the neonatal period. Common clinical features may also include myopathic facies, long fingers and toes, and cryptorchidism (8).Consistent with the neonatal XLMTM cohort reported by Xiong et al. (9), cryptorchidism, generalized hypotonia, and persistent ventilator dependence are the core triad of severe infantile XLMTM. Unlike mild late-onset male patients or female heterozygous carriers, neonatal cases carrying splice-site MTM1 variants universally present with perinatal asphyxia and require prolonged invasive respiratory support. Zídková et al. (10) summarized congenital myopathy cohorts from Central Europe and demonstrated that splice-region MTM1 deletions correlate with the most severe neonatal phenotypes, consistent with the pathogenic variant identified in our proband.

This patient was categorized as small for gestational age and suffered severe neonatal asphyxia, marked by respiratory and circulatory dysfunction at birth, low muscle tone, lack of crying, and bradycardia (11). Severe asphyxia often leads to high mortality rates and can result in persistent ventilator dependence, even in cases where electroencephalogram and cranial ultrasound findings are unremarkable. Differentiating between various causes of hypotonia in neonates requires careful assessment of medical history, family pedigree, existing genetic tests, and laboratory results. Advancements in whole exome sequencing and genomic technologies greatly facilitate the identification of underlying genetic defects and improve diagnostic accuracy for a range of Mendelian disorders (12).

Mutations in the M1M1 gene are a significant contributor to the X-linked recessive inheritance of centronuclear myopathy (13). Located on chromosome Xq28, the M1M1 gene encodes myotubularin 1, crucial for muscle development. Dysfunctional protein expression leads to the disruption of myotubular formation during gestation, resulting in central nucleomyopathy (7). Children with X-linked CNM primarily manifest during the fetal period, often accompanied by increased amniotic fluid, decreased fetal movement, diminished activity, absent tendon reflexes, and, in severe cases, respiratory failure and asphyxia effects (12).Currently, no curative treatments exist for XLMTM, but several promising therapeutic strategies are in clinical trials. The ASPIRO trial utilizing adeno-associated virus gene replacement therapy has shown initial positive outcomes, demonstrating improved muscle strength in mechanically ventilated children under six years old (14). However, complications, including hepatotoxicity leading to mortality, have put such trials on hold. Similarly, a 2018 gene replacement therapy trial involving 24 XLMTM patients demonstrated improved respiratory function but faced suspensions due to severe adverse events (15). Current investigations also explore the efficacy of antisense oligonucleotide therapies, including the Unire-CNM trial, to tackle increased dynamin-2 expression, potentially beneficial in both adult and pediatric patients (16). Additional studies on tamoxifen, which reduces DNM2 levels, are ongoing (7, 16).Regrettably, at this time, there exists no curative treatment for X-linked Centronuclear Myopathy. During the acute phase, it is imperative to maintain respiratory,circulatory, and internal homeostasis (9).

In this case, the child received adjunctive treatments with bromopyrazine and tamoxifen, yet outcomes remained unsatisfactory. XLMTM is characterized by early onset, severe symptoms, and poor prognosis. Early diagnosis, genetic counseling, and prenatal diagnostics are critical for family planning and eugenics. Since hypotonia can manifest in diverse forms, genetic testing is pivotal for accurate diagnosis and management. Families should be well-informed and provided with compassionate care as they navigate this challenging condition.

Prior to this study, the splice site heterozygous deletion variant c.1260 + 2_1260 + 3del (chromosome X: 149826499) discovered in our patients had not been entered into the public gene database. We have submitted this new pathogenic MTM1 variant to the ClinVar database (providing a temporary SUB ID in the paper), and the original raw sequencing data of the entire exome of this infant has been uploaded to the NCBI SRA database, with the BioProject number PRJNA1479542. It is planned to be publicly released on July 31, 2027. This provides a new genotype-phenotype association for the neonatal XLMTM.For families with X-linked central nuclear myopathy, it is strongly recommended to conduct prenatal genetic testing through chorionic sampling or amniocentesis in subsequent pregnancies. Because when the mother is a carrier, there is a 50% chance of passing on the pathological MTM1 mutation to the male offspring. Therefore, some families choose to undergo third-generation *in vitro* fertilization. This case further demonstrates that whole-exome sequencing is an effective tool for diagnosing newborns with low muscle tone, excessive secretions, and continuous dependence on mechanical ventilation with an unclear cause. It can help parents receive genetic counseling as early as possible and accurately assess the risk of conception.

## Data Availability

The datasets presented in this study can be found in online repositories. The names of the repository/repositories and accession number(s) can be found in the article/Supplementary Material.
